# 

**Published:** 1884-08

**Authors:** 


### A Monthly Journal

DEVOTED TO

### DENTAL AND ORAL SCIENCE.

---

W. C. BARRETT, M. D., D. D. S.,

EDITOR.

---

THE NEW YORK DENTAL JOURNAL ASSOCIATION,

PUBLISHERS,

No. 35 West 46th Street, New York.

AND

11 W. Chippewa St., Buffalo, N. Y.

**$2.50 Per Annum in Advance, - - - - Single Copies, 25 Cents.**

Entered at the Post-Office, Buffalo, N. Y., as Second-Class matter.

